# Brazos Valley Medical Association

**Published:** 1896-06

**Authors:** 


### Brazos Valley Medical Association.

The first semi-annual meeting of the Brazos Valley Medical Association was held in Hearne, May 12–13, 1896.

The meeting was called to erder by Dr. H. W. Cummings, of Hearne.

Invocation by Rev. W. W. Homer, of Hearne.

Welcome addresses by Hon. P. L. Brady, Mayor of Hearne; J. L. Hazlett, Commercial Club, and Dr. H. W. Cummings, of Hearne.

Responses to welcome address by Dr. Daniel Parker, Calvert.

Dr. T. A. Pope, of Cameron, was elected temporary chairman; Dr. H. W. Cummings, of Hearne, temporary secretary.

After adoption of constitution and by-laws, nominations for the various offices were in order and the following were elected for the ensuing year:

President, Dr. H. W. Cummings, of Hearne; 1st Vice-President, Dr. Daniel Parker, of Calvert; 2d Vice-Pres., Dr. W. R. Vaughan, of Nesbit; Treasurer, Dr. W. W. Greer, Cameron; Secretary, Dr. E. Brittain, o fBremond.

For Judicial Council—Drs. Geo. R. Tabor, Bryan; F. R. Collard, Wheelock; T. A. Pope, Cameron; J. M. Abney, Franklin; J. W. Hudson, Milano.

Thirty charter members were enrolled. Several interesting papers were read, among them one by Dr. D. H. Bailey, of Branchville, on "Mastoid Abscess," which was freely discussed, and by ballot ordered printed in the TEXAS MEDICAL JOURNAL.

The TEXAS MEDICAL JOURNAL was unanimously selected as the official organ of the Association.

The next meeting of the Association will be held in Bryan on the second Tuesday in November, 1896.

H. W. CUMMINGS, President. E. BRITTAIN, Sec'y.

**The East Texas Medical Association** will hold its regular quarterly meeting at Tyler, on the 14th and 15th July prox. This society is in a prosperous condition and interest in its meetings never flags. Let it be an example to the profession elsewhere.

The work of organizing is going bravely on. It will be a short time only, we hope, before the profession is organized into county and district associations. Then, once a year, let us hold a State Medical Convention—one delegate to so many members, and the real sentiment of the profession on any subject can be gotten at. We got the cart before the horse in organizing a State society before the profession was organized locally, and the voice of the State Medical Society is by no means an accurate echo of the professional sentiment of the State.

